# Furin as a therapeutic target in cystic fibrosis airways disease

**DOI:** 10.1183/16000617.0256-2022

**Published:** 2023-05-03

**Authors:** Lisa E.J. Douglas, James A. Reihill, Bethany M. Montgomery, S. Lorraine Martin

**Affiliations:** School of Pharmacy, Queen's University Belfast, Belfast, Northern Ireland, UK

## Abstract

Clinical management of cystic fibrosis (CF) has been greatly improved by the development of small molecule modulators of the CF transmembrane conductance regulator (CFTR). These drugs help to address some of the basic genetic defects of CFTR; however, no suitable CFTR modulators exist for 10% of people with CF (PWCF). An alternative, mutation-agnostic therapeutic approach is therefore still required. In CF airways, elevated levels of the proprotein convertase furin contribute to the dysregulation of key processes that drive disease pathogenesis. Furin plays a critical role in the proteolytic activation of the epithelial sodium channel; hyperactivity of which causes airways dehydration and loss of effective mucociliary clearance. Furin is also responsible for the processing of transforming growth factor-β, which is increased in bronchoalveolar lavage fluid from PWCF and is associated with neutrophilic inflammation and reduced pulmonary function. Pathogenic substrates of furin include *Pseudomonas* exotoxin A, a major toxic product associated with *Pseudomonas aeruginosa* infection and the spike glycoprotein of severe acute respiratory syndrome coronavirus 2, the causative pathogen for coronavirus disease 2019. In this review we discuss the importance of furin substrates in the progression of CF airways disease and highlight selective furin inhibition as a therapeutic strategy to provide clinical benefit to all PWCF.

## Abstract 2

**This review highlights selective inhibition of furin as a means to regulate aberrant ENaC activity and offers a mutation-agnostic approach to airways rehydration and restoration of mucociliary clearance in cystic fibrosis.** https://bit.ly/3IyDwl4

## Introduction

Cystic fibrosis (CF) is a life-limiting genetic condition that is characterised by progressive lung damage and pulmonary decline. It is driven by chronic cycles of infection and unrelenting inflammatory processes, with symptoms that include a build-up of thick mucus secretions, airways dehydration and impaired mucociliary clearance (MCC) mechanisms. As a result, therapeutic regimens that focus on the treatment of secondary pulmonary complications such as airway obstruction and infection are the mainstay for many people with CF (PWCF) worldwide. CF is, however, caused by mutations in the CF transmembrane conductance regulator (CFTR) gene that encodes for a critical cAMP-dependent chloride channel. The US and EU approval of CFTR modulators for clinical use is, therefore, a significant advancement in the clinical management of CF. A United States Food and Drug Administration approved triple-modulator therapy, elexacaftor–tezacaftor–ivacaftor (ETI) (Trikafta/Kaftrio^®^, Vertex Pharmaceuticals), has increased the population of patients who may benefit from CFTR modulator drugs to just over 90%. Significant therapeutic benefits include an approximate 10% improvement in percentage predicted forced expiratory volume in 1 s (FEV_1_) after 6 months of treatment [1] and a reduction in exacerbations [2] and treatment burden [3]. There is also evidence that ETI has beneficial effects on infection and inflammation [4–6]. However, CFTR modulator treatments are not curative and lung disease associated with continued cycles of infection and inflammation remains [7]. New therapeutic strategies that can be used in tandem with CFTR modulators are therefore required if clinical outcomes in CF are to be further improved. There also exists 10% of PWCF who are not currently eligible for CFTR modulator therapies and who require a mutation-agnostic approach [8]. Importantly, there is also unequal eligibility for CFTR modulator therapies across racial and ethnic minority groups; for example, the number of black and Hispanic PWCF who are not modulator-eligible is two to three times higher than their non-Hispanic white counterparts [9, 10]. As such, there is still a significant need for inclusive treatments for all PWCF.

Furin, a member of the proprotein convertase (PC) family of proteases, is involved in the dysregulation of several key processes that contribute to the pathogenesis of CF airways disease. Here, we provide an overview of a putative role of furin in the progression of CF airways disease and discuss the furin cleavage sites that are present in a wide range of mammalian and pathogenic substrates relevant to disease progression [11]. We propose that the inhibition of furin as a therapeutic strategy not only has the potential to restore airways hydration and rescue MCC, but also may relieve the burden of infection and chronic inflammatory and remodelling processes associated with lung function decline in CF.

## CF airways disease

CF is a life-limiting, autosomal recessive genetic disease that is estimated to affect over 160 000 people worldwide [12], with a predicted median survival age of 50 years [13]. CF is caused by mutations in the gene that codes for the CFTR anion channel, causing disruption of epithelial Cl^-^ and HCO_3_^-^ transport. As CFTR is expressed in a wide range of organs throughout the body, CFTR dysfunction causes multi-organ disease, including lung disease, pancreatic insufficiency, intestinal obstruction, biliary cirrhosis and bilateral absence of the vas deferens. Progressive airways disease is the major cause of mortality and morbidity in PWCF [14]. CF airways disease is characterised by hypersusceptibility and inability to clear infection, alongside mucus obstruction, airway tissue damage and an unrelenting and exaggerated inflammatory response [15]. Lung disease in CF develops early in life, with nearly 60% of children with CF exhibiting bronchiectasis at 3 years old due to chronic cycles of infection and inflammation [16]. The main driving force for the development of bronchiectasis is the presence of increased concentrations of neutrophil elastase (NE), which in addition to causing direct pulmonary damage through degradation of the extracellular matrix, also disrupts innate immunity and increases mucus production [17, 18]. As bronchiectasis progresses, PWCF become increasingly susceptible to infection by a range of pathogens. A common pathogen is *Pseudomonas aeruginosa*, which infects approximately 80% of PWCF over their lifetime and directly contributes to the irreversible decline in pulmonary function observed [19–21].

## Furin in the airways

Furin (PCSK3) is a calcium-dependent, cellular serine endoprotease, which belongs to the PC family of proteases and cleaves after the carboxy-terminal arginine (Arg) residue in the sequence; –Arg–X–Lys/Arg–Arg↓– (where Lys is lysine, X is any amino acid and ↓ identifies the cleavage site) [22]. Furin is expressed ubiquitously in mammalian tissues, including in airway epithelial cells (AECs), with evidence that furin is localised in ciliated cells in well-differentiated AEC models [23]. Within the cell, furin localises to the trans-Golgi network (TGN). From there, furin can traffic through several endosomal compartments to the cell surface [24] where it can be tethered by actin-binding protein-280 or, alternatively, a truncated form of furin may be shed from the cell surface [22, 25, 26].

Furin has broad and important roles in ensuring cellular homeostasis, with furin cleavage sites present in a wide range of mammalian proteins, including growth factors, cytokines, metalloproteinases, receptors and membrane channels as well as bacterial toxins and viral glycoproteins [27]. Furin has been reported to be present at elevated levels in CF AECs in comparison to non-CF AECs and many substrates of furin have key roles in the pathogenesis of CF airways disease [28, 29]. It is, therefore, suggested by these *in vitro* studies that furin levels may be elevated in CF airways and contribute to disease progression. As such, approaches to normalise increased furin activity offer an attractive therapeutic strategy.

## Furin as a regulator of the epithelial sodium channel (ENaC)

In healthy airways, a thin film of fluid known as airway surface liquid (ASL) regulates MCC – a critical component of innate immune defence, facilitating the removal of inhaled pathogens [30]. The ASL layer is composed of two layers, the periciliary liquid (PCL) layer, which consists principally of salt and water and “bathes” the cilia, and the mucus gel layer, which comprises mucin glycoconjugates [31, 32]. As the mucus layer also has a high water content, it may serve as a “reservoir” to donate water to the PCL as required [33].

ASL volume is regulated by the net osmotic gradient established by oppositely directed Cl^-^ and Na^+^ flux across the airway epithelium [34]. In CF airways, however, CFTR is dysfunctional or absent and therefore fails to secrete Cl^-^ into the airway lumen. Secondary to this loss of functional CFTR, the CF epithelium displays unrestrained Na^+^ absorption *via* ENaC [35–38], which subsequently leads to a depletion of PCL volume, causing the collapse of a viscous mucus layer onto the surface of the airway epithelium. This leads to the “collapse” of cilia and diminished mucus transport, which reduces the airways’ ability to clear inhaled pathogens and predisposes the individual to cycles of chronic infection and inflammation [39]. Thus, increased ENaC-mediated Na^+^ and associated fluid transport across the airway epithelium play critical roles in the pathogenesis of CF airways disease.

The β-ENaC overexpressing transgenic mouse develops a severe spontaneous lung disease that shares key features of CF associated with an increase in mortality, including mucus obstruction, neutrophilic inflammation and infection [40]. Knockout of the ubiquitin ligase Nedd4–2 in lung epithelia of mice also causes a similar CF-like lung disease linked to enhanced ENaC function [41]. In pseudohypoaldosteronism, where patients have loss-of-function mutations in the ENaC, there are markedly increased airways hydration and MCC rates [42]. Similarly, a group of CF patients with lowered ENaC activity due to a mutation in the δ-subunit exhibit a less progressive disease [43]. The mouse models and human studies together demonstrate the importance of ENaC in maintaining homeostatic airways hydration and show an important link between increased ENaC activity and the development of a CF-like pulmonary disease.

### Furin proteolysis primes ENaC for activation

ENaC exhibits low conductance for Na^+^ in the absence of proteolysis and is activated through cleavage of its α and γ subunits [44]. For a proportion of ENaC, the activation process starts when it passes through the biosynthetic pathway [45, 46]. For this distinct pool, furin cleaves the α subunit at two sites, releasing a small inhibitory peptide from the extracellular loop, partially activating the channel and cleaving γ-ENaC at a single position. These actions are thought to “prime” ENaC for further cleavage at a secondary site on the γ subunit by cell-surface channel-activating proteases (CAPs) such as prostasin or soluble proteases including trypsin and NE [47–49] and bacterial proteases [50]. Furin activity is, therefore, seminal to the proteolytic regulation of ENaC and, as a consequence, is a key factor that contributes to the hydration status of the airway epithelium.

### The effect of protease inhibitors on ENaC activity

**FIGURE 1:**
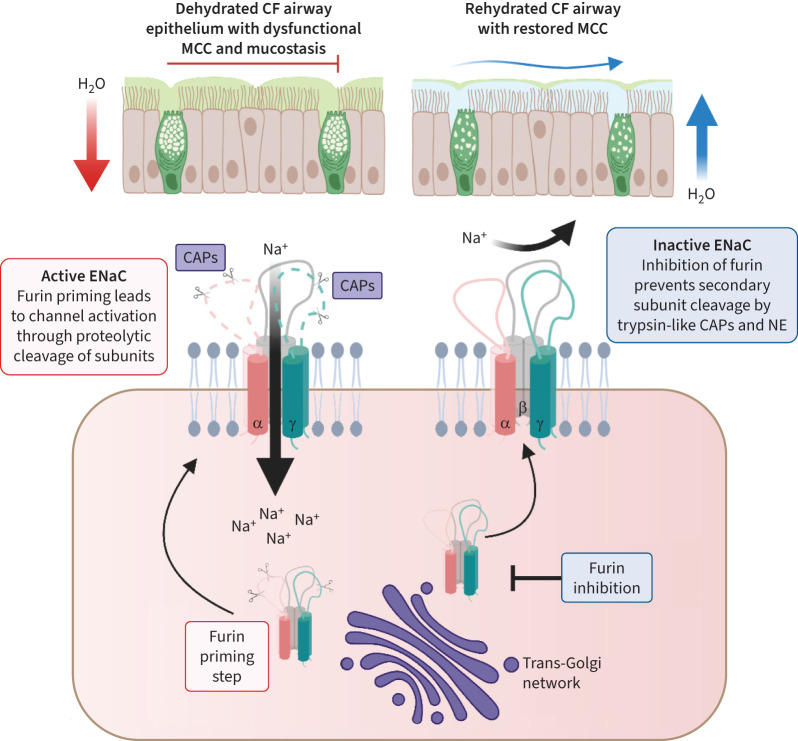
Inhibition of the epithelial sodium channel (ENaC) leading to airways hydration and restored mucociliary clearance. Furin cleaves a pool of ENaC as it passes through the biosynthetic pathway, priming ENaC for further cleavage at the cell surface by channel activating proteases (CAPs). In cystic fibrosis (CF), loss of functional cystic fibrosis (CF) transmembrane conductance regulator and hyperabsorption of Na^+^ by ENaC causes dehydration of the airway surface and collapsed mucociliary clearance (MCC). Inhibition of furin prevents secondary proteolytic cleavage of ENaC subunits by cell surface trypsin-like CAPs as well as neutrophil elastase (NE), present in high levels in CF airway secretions. Inhibition of ENaC-mediated Na^+^ absorption increases airway surface hydration and restores mucociliary transport. Created with BioRender.com.

The direct inhibition of CAPs as a therapeutic strategy in CF has included the use of broad-spectrum inhibitors of trypsin-like proteases (TLPs) such as aprotinin and camostat mesylate, both of which were observed to attenuate ENaC function *in vitro* and *in vivo* [51–53]. While the inhibition of extracellular TLPs has clear therapeutic potential, there are a number of challenges. In particular, evidence shows that the effect of broad-spectrum inhibitors, such as that of aprotinin on ENaC, can be overwhelmed by the array of active proteases present within the microenvironment of the CF lung [54, 55]. Furin inhibition, however, offers an alternative strategy (figure 1). Decanoyl-RVKR-CMK (Fur I) and QUB-TL1, both inhibitors of trypsin-like CAPs and furin, have been shown to inhibit ENaC-mediated Na^+^ absorption [29, 55]. In addition, these compounds afford protection against subsequent NE-mediated ENaC activation; levels of active NE are significantly elevated in the CF airway, suggesting that furin inhibition may offer additional benefits over the direct targeting of TLPs. We have further demonstrated that highly selective and potent inhibition of furin by a first-in-class cell-permeable compound (BOS-318) caused a sustained suppression of ENaC activity in CF AECs that resulted in increases in ASL height and mucociliary transport rates [54]. Importantly, BOS-318 was also observed to afford protection against ENaC activation mediated by both NE alone and the complex mix of active proteases contained within purulent CF sputum sol [54]. Uniquely, BOS-318 shows high selectivity for furin and does not directly inhibit any of the extracellular CAPs known to inhibit ENaC, attributed to the unique binding mode of BOS-318 that does not engage the active site catalytic serine of furin [55]. This highlights the importance of furin in ENaC regulation and reinforces selective furin inhibition as a superior therapeutic avenue compared to the broad spectrum targeting of CAPs [29, 54].

## Other key roles for furin in CF airways disease pathogenesis

**FIGURE 2:**
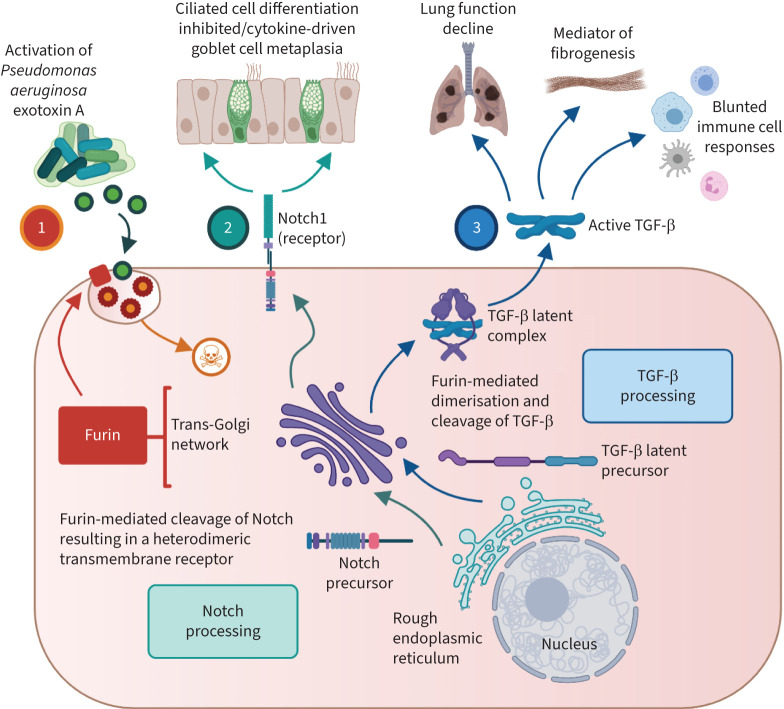
Additional multi-factorial benefits of inhibiting furin in cystic fibrosis**.** Furin-mediated processes include, but are not limited to 1) activation of cytotoxins such as *Pseudomonas aeruginosa* exotoxin A [56], 2) processing of Notch1 which regulates basal cell fate through inhibition of ciliated cell differentiation and by facilitating a cytokine-driven goblet cell metaplasia [57–59], and 3) induction of transforming growth factor-β (TGF-β) processing leading to higher levels of active TGF-β, which is generally associated with lung function decline [60–63], fibrogenesis and a suppression of immune cell responses [64, 65]. Created with BioRender.com.

Furin has been implicated in a number of other regulatory pathways associated with disease pathogenesis in CF to include activation of bacterial exotoxins, Notch signalling and activation of transforming growth factor beta (TGF-β), as summarised in figure 2 and described in detail below.

### Furin as a regulator of bacterial infection in the airways

Several bacterial and viral pathogens (or their toxins) require processing by PCs including furin in order to exert their harmful effects. One such bacterial toxin is *Pseudomonas* exotoxin A (PE), secreted by *P. aeruginosa*. Furin has been implicated in the cleavage of PE to form a 37 kDa C-terminal fragment that translocates to the cytosol of host cells where it inhibits protein synthesis through inactivation of cytosolic elongation factor 2, causing cell death [56, 66]. Additionally, there is evidence that furin may also influence the quantity of the toxin receptor on target cells [67]. Chronic infection with *P. aeruginosa* occurs in over 20% of adults with CF in the UK, with intermittent infection occurring in a further 20% [68], presenting a major cause of mortality and morbidity. Infection with PE-producing *P. aeruginosa* is associated with an elevated mortality rate in comparison to non-PE-producing strains [69] and increasing levels of serum IgG antibodies to PE is associated with poor prognosis [70]. PE is also detected in the respiratory secretions of PWCF and has been shown to contribute to mortality in experimental animals [71, 72]. While nonselective inhibitors of furin such as decanoyl-RVKR-CMK and QUB-TL1 demonstrated a reduction in PE-induced cytotoxicity in CF AECs [29], similar studies using a highly selective furin inhibitor, BOS-318, have confirmed the role of furin in the PE intoxication process and adds further weight to the therapeutic potential of furin inhibition in the airways [54].

### The role of furin and Notch signalling in cell fate choices in the airways

Notch signalling has vast and complex roles within pulmonary development and regeneration, including in cell fate choices as well as in cell turnover and repair [73]. Members of the family of Notch receptors (Notch1–4) that are expressed in the airways have a furin cleavage event as the first step in their proteolytic activation process.

Notch precursors are cleaved at the S1 site during maturation in the TGN by a furin-like convertase, producing a heterodimeric receptor located at the cell surface [74]. The key role that furin processing plays in Notch signalling was confirmed by knocking out furin activity, which was found to strongly reduce Notch1 cleavage and by the mutation of two furin sites on Notch1 that completely impaired Notch signalling [59]. There is also evidence of a positive feedback loop between furin and Notch1, where Notch1 can drive furin expression, thereby increasing the activities of ADAM metallopeptidase domain 10 and membrane-type matrix metalloproteinase 1, both of which cleave furin-processed Notch at the cell surface, in turn further increasing Notch1 signalling [75].

Hypersecretion of mucus and reduced MCC are clinical characteristics of CF airways disease. In both the developing and adult airway epithelium, notch signalling plays a critical role in the differentiation of basal progenitor AECs into secretory or ciliated cell types. Additionally, although active at steady state, it increases during epithelial repair processes [76]. Evidence gathered using human airway epithelium showed that Notch agonist treatment caused an increase in the number of mucus-producing cells and a reduction in ciliated cell number, with Notch antagonists having the opposite effect and preventing interleukin (IL)-13-driven mucus metaplasia [77]. Several studies have further probed the individual roles of the Notch family members (Notch1–4) in cell fate decisions in the airway epithelium. The blocking of Notch2 signalling by a Notch2-specific blocking antibody was shown to reduce the expression of goblet cell markers while simultaneously increasing the expression of ciliated cell markers in a three-dimensional “bronchosphere” cell model. Further, blocking Notch2 inhibited IL-13-driven goblet cell metaplasia and partially restored ciliated cells [57]. This study also reported that Notch1 or Notch3 signalling were not required in basal cell fate decisions to differentiate to a ciliated or secretory cell type. In contrast to these findings, a study performed in differentiated human airway cultures found that sustained expression of the intracellular domain of Notch1 and Notch3 skewed the differentiation of cells to a secretory cell fate with a concomitant decrease in the differentiation of ciliated cells [58].

#### Dysregulated Notch signalling and COPD

Notch signalling dysregulation has been linked to several respiratory diseases, including COPD. A study that investigated Notch signalling in the context of rhinovirus-induced goblet cell hyperplasia in COPD using differentiated human airways cultures found that inhibiting Notch3 but not Notch1, by short hairpin RNA specific to Notch3 or Notch1 lentivectors, attenuated goblet cell hyperplasia, reducing goblet cell numbers [78]. Expression of Notch3 and the Notch ligand, DLL1 (delta-like canonical Notch ligand 1), were downregulated in small airway epithelia cells from COPD donors in comparison to nonsmoking donors [79]. A subsequent study using immunofluorescence demonstrated that human COPD bronchial tissue has enhanced NICD1 (Notch1 intercellular domain) and HEY2 (hairy/enhancer-of-split related with YRPW motif protein 2) (both key in Notch signal transduction) in areas of goblet cell metaplasia in the airway epithelium and submucosal glands [80]. Further, cigarette smoke extract (CSE) has been demonstrated to induce Notch3 signalling and mucin 5AC^+^ (MUC5AC^+^) goblet cell differentiation in cultured human bronchial epithelial cells, with small interfering RNA-mediated Notch3 knockdown found to supress the effect of CSE on MUC5AC expression [81].

#### Dysregulated Notch signalling and CF

In CF, enhanced MUC5AC and depleted β-tubulin expression have been observed in the airways, suggesting the presence of a decreased ciliated cell to goblet cell ratio [82]. As Notch signalling is regulated by furin cleavage, furin inhibition could drive cell fate decisions away from mucus-producing cell types towards ciliated cell types, with correction potentially able to contribute to the restoration of effective MCC. Indeed, this hypothesis was tested in a study using a broad-spectrum inhibitor of furin, decanoyl-RVKR-CMK, which demonstrated that treatment of human nasal epithelial cells with this inhibitor showed reduced Notch1 processing and enhanced ciliated cell differentiation. Knockdown of furin in these cells also caused a reduction in Notch1 processing and an increase in ciliated cell numbers, further demonstrating the involvement of furin in this process [83].

### Regulation of TGF-β by furin

TGF-β, of which there are three isoforms (TGF-β1, 2 and 3, with TGF-β1 being the most widely studied), is initially synthesised as a pre–pro form that includes a signalling peptide, a covalently attached large N-terminal portion called the latency-associated peptide (LAP) and a short C-terminal segment corresponding to the active form of TGF-β. Within the TGN, furin has been reported as the principal PC responsible for proteolytic cleavage of LAP from the mature, biologically active dimeric TGF-β protein [84]. LAP, however, remains noncovalently attached as part of the small latent TGF-β complex, which prevents the binding of TGF-β to its receptors and maintains latency. Binding of a third protein, latent TGF-β-binding protein, through a disulphide linkage to one of the chains of the LAP/TGF-β dimer comprises the large latent complex, which is subsequently secreted from cells and processed extracellularly to release active TGF-β. Evidence shows that other members of the PC family may function to substitute for or supplement furin activity in the TGN [61, 62]. Interestingly, TGF-β1 can upregulate the gene expression of furin, resulting in increased pro-TGF-β1 processing, suggesting that furin and TGF-β1 exist in a positive feedback loop [28, 85].

TGF-β is expressed by alveolar macrophages as well as airway epithelia and smooth muscle cells and has many important roles in maintaining homeostasis in the human airway [86]. Increased TGF-β signalling has been described in a number of pulmonary diseases including idiopathic pulmonary fibrosis [87] and COPD [88, 89]. In CF, total TGF-β1 has been observed in increased quantities in the bronchoalveolar lavage fluid of PWCF in comparison to non-CF controls with levels associated with neutrophilic inflammation, diminished lung function as determined by FEV_1_ and recent hospitalisation [63]. In other studies, increased TGF-β1 levels in plasma was associated with *P. aeruginosa* infection and lower FEV_1_ values in PWCF [90, 91]. Levels were also found to increase during exacerbation and reduce after antibiotic therapy [92]. The importance of TGF-β in CF is further demonstrated by studies which have shown a linkage between two polymorphisms (−509 T in the promoter region and T29C in codon 10) and increased disease severity [60, 93]. TGF-β also exerts a number of effects on ion transport, airway remodelling and inflammation relevant to CF airways disease pathogenesis, which will be discussed in turn.

#### Effect of TGF-β on ion transport

TGF-β can affect CFTR expression and function and thus can indirectly modulate ion transport in the airways. Several studies performed on colonic epithelial cells showed that TGF-β can decrease both chloride secretion and CFTR protein expression [94, 95]. Similarly, studies using primary differentiated human bronchial epithelial cells and non-CF and CF mice, observed that CFTR mRNA levels were found to be reduced with TGF-β1 treatment [96, 97]. Additionally, CF AECs treated with TGF-β1 showed reduced VX-809 (CFTR corrector, lumacaftor) mediated rescue of F508*del*-CFTR [97]. There is also evidence that TGF-β plays a regulatory role in other chloride channels, such as calcium-activated chloride channels (CaCCs). TGF-β treatment of non-CF AECs reduced both CaCC and CFTR-dependent chloride currents and caused a reduction in CFTR and transmembrane member 16A (TMEM16A) protein levels. Additionally, ASL height regulation in these AECs was disrupted after TGF-β treatment [98]. A proposed mechanism for the observed effects of TGF-β on chloride transport is through regulation of microRNAs (miRs), such as miR-145, which is upregulated by TGF-β and can bind directly to the CFTR mRNA transcript, causing decreased CFTR protein synthesis. miR-145 has been found to be increased in the bronchoalveolar lavage of PWCF in comparison to non-CF samples [99, 100].

In addition to the regulation of chloride secretion, TGF-β may also modulate the activity of other ion channels within the airways. In a study where non-CF and CF mice were treated with an adenovector containing TGF-β1 cDNA, whole-lung ENaC mRNA was increased in both genotypes in comparison to control animals, suggesting that TGF-β1 could have an upregulatory effect on ENaC [96]. In human AECs, apically expressed large-conductance, Ca^2+^-activated and voltage-gated K^+^ (BK) channels can influence airway hydration and MCC by providing an electrochemical gradient for Cl^−^ secretion [101]. TGF-β has been shown to modulate these BK channels, causing decreased ASL volume and ciliary beat frequency, an effect which can be reversed by a clinically used TGF-β signalling inhibitor, pirfenidone [102]. Rescue of BK channel function by the anti-inflammatory drug losartan was also observed to improve airway hydration in CF AECs and MCC in a sheep model of CF [103].

#### Effect of TGF-*β* on airway remodelling

TGF-β has other putative roles in the pathogenesis of CF airways disease. The production of collagen, the most abundant component of the extracellular matrix, is directly stimulated by TGF-β [104]. Prominent TGF-β signalling in lung tissue correlates with increased myofibroblast differentiation, fibrosis [105] and thickening of the basement membrane [106]. Total soluble collagen levels have also been found to be increased in CF cell lines in comparison to non-CF controls, which were decreased both by TGF-β-blocking antibodies and a furin inhibitor, alpha-1 antitrypsin Portland variant [28]. Augmented TGF-β signalling may therefore contribute to aberrant lung remodelling in CF.

#### Effect of TGF-*β* on inflammation

Chronic neutrophilic inflammation is a characteristic of CF airways disease. TGF-β has been shown to stimulate neutrophil chemotaxis [107]. Further, NE, which is secreted by neutrophils and present in elevated concentrations in the CF lung, has been shown to stimulate the secretion of TGF-β in airway smooth muscle cells [108]. Additionally, NE-null mice showed reduced TGF-β activation in response to bleomycin treatment and resistance to bleomycin-induced fibrosis [109]. In CF airways disease, a positive correlation between sputum TGF-β levels and levels of pro-inflammatory cytokines IL-1β and IL-8 was observed [91]. As both of these cytokines are present in increased concentrations in the CF lung, this provides some evidence to suggest that TFG-β also plays a role in chronic inflammatory processes in CF [110]. This is impacted further by the contribution of TGF-β to the persistence of some bacterial pathogens through the suppression of innate immune responses [65], to include impaired bacterial killing by CF macrophages [111]. TGF-β-treated peripheral blood monocyte-derived macrophages (MDMs) have been shown to have a two times reduced ability to kill *P. aeruginosa.* Furthermore, a reduced antimicrobial ability of MDMs was observed when co-cultured with primary CF AECs in comparison with non-CF controls. An enhanced *P. aeruginosa* killing ability of MDMs in CF AEC co-culture was, however, restored to the levels of MDM/non-CF AEC co-culture upon treatment with TGF-β blocking antibodies and furin inhibitor decanoyl-RVKR-CMK [28].

A link between enhanced furin activity, increased TGF-β levels and both enhanced soluble collagen production and reduced anti-pseudomonal activity by CF AECs therefore exists. Additionally, highly selective furin inhibition has been shown to have beneficial effects on overactive ENaC, ASL height and mucociliary transport in CF AECs [54]. However, further investigation is required to determine whether TGF-β modulation contributes to these effects and, indeed, whether other pathogenic features of CF associated with increased TGF-β signalling such as neutrophil chemotaxis can be modulated by furin inhibition.

## The therapeutic potential of furin inhibition beyond CF airways disease

In addition to the role of furin in CF, the cleavage of host, bacterial and viral protein substrates by furin is implicated in the pathogenesis of a number of other conditions, which highlights the broader utility of highly selective furin inhibition as a therapeutic.

### Furin and COPD

COPD shares many clinical manifestations with CF, including increased mucus secretion, airways dehydration leading to loss of effective MCC and chronic inflammation. While the aetiology of the two diseases differ, CFTR activity is also negatively impacted in COPD with acquired CFTR dysfunction associated with exposure to cigarette smoking [112–115]. This reduction in CFTR function leads to augmented ENaC activity, contributing to disease pathogenesis [116]. Further, furin expression has been found to be elevated in the small airway epithelium of COPD patients and in smokers [117]. As such, furin inhibition also presents a viable therapeutic target in COPD.

### Furin and cancer

Dysregulation of furin expression and activity can correlate with the progression and aggressiveness of a number of different types of cancer, including colon carcinoma, nonsmall cell lung carcinoma, breast cancer and head and neck cancers. Furin has been targeted clinically in the treatment of some cancers by an autologous cancer cell vaccine partly based on the silencing of furin. Vaccine manufacturing involves harvesting cells from patients and transfecting *via* electroporation extracorporeally with a plasmid encoding granulocyte–macrophage colony-stimulating factor and a bifunctional short hairpin RNAi targeting furin [118]. This treatment has been assessed in phase I and II clinical trials and showed benefit in advanced ovarian cancer [118], Ewing sarcoma [119] and hepatocellular carcinoma [120], indicating that furin repression has therapeutic potential in the treatment of some types of cancer.

### Furin and infectious disease

Furin also has putative roles in a number of infections through its cleavage of several bacterial and viral substrates. Bacterial substrates include anthrax toxin, diphtheria toxin and PE as previously discussed [121]. Furin also enables cellular entry of several pathogenic viruses belonging to evolutionarily diverse families [122], including severe acute respiratory syndrome coronavirus 2 (SARS-CoV-2), the causative pathogen for coronavirus disease 2019, where furin cleaves a site critical for virus–host cell fusion and entry into lung cells, between the S1/S2 subunits of the spike (S) glycoprotein [123, 124]. Indeed, a number of furin inhibitors have been investigated against SARS-CoV-2, including the nonselective inhibitor decanoyl-RVKR-CMK, which blocked viral entry and decreased viral production of SARS-CoV-2 [125], and the synthetic furin inhibitor MI-1851, which inhibited viral replication [126]. Further, highly selective furin inhibitors, including the previously discussed BOS-318, blocked S protein processing and prevented viral infection of Calu-3 cells when combined with serine protease inhibitor, camostat [127].

## Challenges of furin as a therapeutic target

While there have been numerous reports of potent inhibitors of furin [128], the development of highly selective inhibitors has remained a challenge, largely due to the high level of conservation between the substrate binding domain of furin and that of other PCs. For example, the widely used inhibitor, decanoyl-RVKR-chloromethylketone (Fur I), inhibits other PCs with similar potency to furin [54] and also inhibits proteases from other classes, to include TLPs [129]. This high level of conservation means that redundancy exists between furin and other related PCs. Therefore, in order to achieve efficacy and limit the potential for off-target effects, therapeutic interventions must aim to specifically target the PC or PCs driving the relevant pathogenic processes. The development of first-in-class, highly selective inhibitors that target furin with greater potency than other PCs [54, 127] offers an enhanced opportunity to explore the role of furin in the pathogenesis of disease.

Even with advancements in the development of selective inhibitors of furin, the ubiquitous expression of furin and its requirement for the activation of cellular substrates involved in homeostatic processes has raised concerns regarding the potential for off-target effects. Indeed, complete furin knockout in mice leads to embryonic lethality [130], suggesting that long-term, systemic administration of a furin inhibitor may lead to toxic effects. However, studies have shown that liver-selective furin knockout in mice is not lethal and furin substrates continued to be processed, albeit with lesser efficiency, suggesting that some level of redundancy between furin and other PCs was maintained in this model [131]. Moreover, the conditional deletion of furin in T-cells still allowed the cells to undergo normal thymic development; however, it also resulted in loss of peripheral tolerance suggesting the presence of limited redundancy for furin in some processes [132]. This reinforces the concept that local administration of a furin inhibitor directly into the airways, in the case of CF or COPD, could limit systemic exposure and reduce the risk of off-target effects to other organ systems. Further clinical development of a furin inhibitor for the treatment of chronic airways disease will, however, have to remain cognisant of the potential level of redundancy that may exist between furin and other PCs in the airways when determining the safety and efficacy of locally targeted furin inhibition in diseases such as CF.

## Conclusions

Treatment of CF airways disease has advanced significantly with the introduction of the highly effective modulator treatment, Trikafta/Kaftrio. There does, however, remain approximately 10% of PWCF for whom there is currently no suitable CFTR modulator drug regimen and who may benefit from a mutation-agnostic treatment such as therapeutics targeting ENaC. For PWCF who are suitable candidates for modulator therapies, there is potential for treatments which modulate ENaC to be used in combination with CFTR-modulating drugs to further improve clinical outcomes. When ENaC is inhibited and Na^+^ absorption is reduced, the apical membrane of the AEC becomes hyperpolarised, providing an increased electrical driving force for Cl^-^ secretion *via* CFTR [133, 134], suggesting that the two approaches may work synergistically. Furin activity has been reported to be elevated in airways cells from PWCF [28] and highly selective furin inhibition has been shown to significantly and durably suppress ENaC activity [54]. This reinforces furin inhibition as an attractive therapeutic strategy in CF, which may “normalise” furin levels and increase the efficacy of existing CFTR modulators.

In conclusion, furin offers an attractive therapeutic target to target airways dehydration in CF caused by loss of CFTR-mediated chloride secretion and the resulting unrestrained ENaC activity as well as other key aspects of the disease, including infective, inflammatory and remodelling processes. Furin inhibition may offer a therapeutic option for PWCF for whom there is no suitable CFTR-modulating therapy available and, importantly, has potential to be used in combination with existing CFTR modulator regimens to improve clinical benefit for PWCF.

**Questions for future research**

- Can selective furin inhibition be sufficiently localised to the airways to prevent off-target effects, such as hyperkalaemia in the renal system?
- Will the benefits observed in *ex vivo* models of CF airways disease be translated to CF animal models and ultimately show benefit in clinical trials?
- Will increases in airways hydration and MCC as a result of ENaC inhibition translate to clinical benefit in all people with CF?
- Will additional potential benefits of furin inhibition, such as protection against the activation of bacterial toxins and suppression of TGF-β signalling, provide a cumulative clinical benefit in CF?
